# YTHDC1 aggravates high glucose-induced retinal vascular endothelial cell injury via m6A modification of CDK6

**DOI:** 10.1186/s13062-024-00498-7

**Published:** 2024-07-08

**Authors:** Qi Zhou, Min Tian, Yang Cao, Min Tang, Xiaohong Xiang, Lu Guo, Hongbin Lv

**Affiliations:** Department of Ophthalmology, Affiliated Hospital of Southwest Medical University, Luzhou City, Sichuan Province 646000 China

**Keywords:** YTHDC1, m6A, Retinal vascular endothelial cell, CDK6

## Abstract

**Objective:**

Retinal vascular endothelial cell (RVECs) injury is a major cause of morbidity and mortality among the patients with diabetes. RVECs dysfunction is the predominant pathological manifestation of vascular complication in diabetic retinopathy. *N*6-methyladenosine (m6A) serves as the most prevalent modification in eukaryotic mRNAs. However, the role of m6A RNA modification in RVECs dysfunction is still unclear.

**Methods:**

RT-qPCR analysis and western blot were conducted to detect the change of m6A RNA modification in diabetic retinopathy. CCK-8 assay, transwell experiment, wound healing assay, tube formation experiment, m6A-IP-qPCR were performed to determine the role of YTHDC1 in RVECs. Retinal trypsin digestion test and H&E staining were used to evaluate histopathological changes.

**Results:**

The levels of m6A RNA methylation were significantly up-regulated in HG-induced RVECs, which were caused by increased expression of YTHDC1. YTHDC1 regulated the viability, proliferation, migration and tube formation ability in vitro. YTHDC1 overexpression impaired RVECs function by repressing CDK6 expression, which was mediated by YTHDC1-dependent mRNA decay. Moreover, it showed sh-YTHDC1 inhibited CDK6 nuclear export. Sh-YTHDC1 promotes the mRNA degradation of CDK6 in the nucleus but does not affect the cytoplasmic CDK6 mRNA. In vivo experiments showed that overexpression of CDK6 reversed the protective effect of sh-YTHDC1 on STZ-induced retinal tissue damage.

**Conclusion:**

YTHDC1-mediated m6A methylation regulates diabetes-induced RVECs dysfunction. YTHDC1-CDK6 signaling axis could be therapeutically targeted for treating DR.

## Introduction

With effective prevention and adjustment of dietary habits, the incidence of diabetes is decreasing year by year. While diabetic retinopathy (DR) is the most common ophthalmic microangiopathy [1]. Diabetic retinopathy is mainly caused by retinal vascular dysfunction and endothelial cell damage induced by long-term high glucose microenvironment, which eventually leads to vision loss and even blindness [2]. It is reported [3] that the number of people with diabetic retinopathy is expected to increase to 642 million by 2040, mainly influenced by age and genetic factors. Visual impairment caused by DR greatly affects the quality of life of patients, so the prevention and treatment of DR has become a global public health problem, and the elucidation of its complex molecular mechanism is extremely urgent, attracting the attention of ophthalmologists in particular.

Retinal vascular endothelial cells have been reported to be a major participant in diabetic retinopathy, and the imbalance of cellular metabolism may lead to inflammatory responses [4], abnormal proliferation and angiogenesis [5]. Retinal vascular endothelial cells were exposed by continuous oxidative stress [6], advanced glycation end products, and inflammatory factors in high glucose environments. Vitamin D regulate the production of advanced glycation end products and prevent the translocation of nuclear factor Kappa B, which could mitigate the damage of oxygen stress to retinal vascular endothelial cells and relieve the progression of DR [7]. Xue et al. found [8] that FTO affected the function of endothelial cells by modulating CDK2 mRNA stability in an m6A-YTHDF2-dependentmanner. Their study suggested that FTO plays an important role in endothelial function and retinal homeostasis in patients with diabetic retinopathy and warrants further investigation as a therapeutic target for patients with DR. It has been reported [9] that HAGLR can be modified by IGF2BP2 in an m6a dependent, as sponge of miR-106-5p, to further activate the PTEN/Akt signal pathway, intensify the development of diabetic retinopathy. Therefore, studies focusing on the function of vascular endothelial cells are gradually increasing, and elucidating the molecular mechanism of endothelial cells under high glucose microenvironment may become a new strategy for the treatment of diabetic retinopathy.

Post-transcriptional regulation of mRNA helps cells cope with the effects of external stimuli and mechanical stress. N6-methyladenosine (m6A) is the most abundant reversible modification in eukaryotic cells and is considered to be a key post-transcriptional regulation of mRNA. M6A methylation includes writers, readers, and Erasers, which indirectly affect transcription, stabilization, and translation. As a reader, YTHDC1 recognizes m6A modified XIST and promoted XIST-mediated transcriptional silencing on X-chromosome [10]. A recent study [11] showed that YTHDC1 promotes exon inclusion of targeted transcripts by selectively recruiting the pre-mRNA splicing factor SRSF3 while blocking the binding of SRSF10 mRNA. In addition, previous studies have shown that m6A methylation is associated with the progression of various diseases, especially cancer [12], cardiovascular diseases [13] and even metabolic diseases [14]. However, the molecular mechanism of YTHDC1-mediated methylation modification in diabetic retinopathy has not been reported.

In the present study, we examined how high glucose affect various gene expression and m6A methylation in rat retinal vascular endothelial cells. We hypothesized that silencing YTHDC1 inhibits CDK6 methylation and affects the invasion, migration and tubeforming ability of vascular endothelial cells. Our study may reveal that YTHDC1 mediated methylation may be a novel strategy for the treatment of diabetic retinopathy.

## Materials and methods

### Cell culture and vectors transfection

Primary rat retinal vascular endothelial cells were isolated from rat retina. The cells were cultured in the Dulbecco’s modified Eagle’s medium (DMEM, Gibico, USA) containing 10% fetal bovine serum. The cells were then put into the incubator with humidified atmosphere containing 5% CO_2_ at 37 °C. The small interfering RNA for YTHDC1 was obtained from RiboBio (Guangzhou, China). The cDNA fragments for YTHDC1 were amplified and cloned into pcDNA3.1 vectors to obtain overexpressed vectors for YTHDC1 (OE-YTHDC1), which were constructed by Sangon Biotech (Shanghai, China). Finally, the Lipofectamine 3000 transfection kit (Invitrogen, USA) was employed to deliver all the above vectors into RVECs according to the manufacturer’s instruction. NG, normal glucose; HG, high glucose.

### Diabetic retinopathy model

The study was approved by the Animal Ethics Committee of West China Hospital, and all experiments are conducted in accordance with guidelines for the use and care of laboratory animals, following the principles of reducing animal numbers and suffering. Diabetic retinopathy rats were induced by intraperitoneal injection of 65 mg/kg STZ (streptozotocin, Sigma, USA). The SD rats were fasted for 12 h before injection. Then, STZ was dissolved in 10 mM citrate buffer (pH = 4.5) and injected continuously for 7 days to induce DR model. At the same time, the sham rats were intraperitoneally injected with sodium citrate buffer. After the last injection, blood was collected from the tail vein for blood glucose detection. A blood glucose meter was used to detect blood glucose concentration greater than 16.7 mmol/L for consecutive seven days, indicating that the diabetes model was successful. Besides, fasting blood glucose was monitored and recorded every other week after the end of injection.

### Intravitreal injection of AAV

According to the previously reported method [15], AAV overexpression plasmid was injected intravitreally into male SD rats (230 ± 10 g) a week before induction of diabetic retinopathy. Based on the promoter ITR, sh-YTHDC1 and CDK6 sequences were cloned into AAV9 vector and packaged into adeno-associated virus (AAVs) by General Biotech. Approximately 1 µL AAV9 was injected into the vitreous cavity with the needle directly above the optic nerve head. Six weeks after diabetes model successfully, the retina tissue were collected for subsequent experiments.

### Hematoxylin and eosin (H&E) staining

Retinal tissue was fixed with 4% neutral formalin and embedded in paraffin. Sections were stained with H&E as per normal procedure [16]. Histopathological changes were analyzed professionally by pathologists who were not previously aware of the groups.

### Retinal trypsin digestion assay

Retinal trypsin digestion was detected using the previously reported method [17]. Retinal vessels were stained with periodic acid-Schiff-hematoxylin and observed and photographed under a light microscopy.

### M6A sequencing (m6A-seq)

Total RNA was isolated from RVECs using TRIzol reagent (Invitrogen, USA) according to the manufacturer’s protocol. In the study, RNA immunoprecipitation was performed with an m6A-specific antibody. The m6A RNA-seq service was provided by Shanghai Oebiotechnology Co., LTD. (Shanghai).

### Real-time qPCR

The TRIzol kit (Invitrogen, USA) was employed to extract the total RNA from RVECs cells according to its protocol. The iScript cDNA Synthesis Kit (Bio-rad, USA) was used to reversely transcribed the RNA into cDNA, and HiScript II Q Select RT SuperMix (Vazyme, China) was employed to quantify the expression status of the target genes. GAPDH was used as an internal control.

### Western blotting assay

The RIPA lysis buffer (Beyotime, China) was used to extract the total proteins from the RVECs cells according to the manufacturer’s protocol. The protein concentrations were determined by using the BCA protein assay kit (Beyotime, China). After that, the proteins were separated by 10% SDS-PAGE and transferred onto PVDF membranes (Millipore, USA). The PVDF membranes were then blocked by 5% skim milk for 60 min at room temperature and probed with the primary antibodies against GAPDH (Abcam, UK), METTL3 (Abcam, UK), METTL14 (Abcam, UK), WTAP (Abcam, UK), ZC3H13 (Abcam, UK), KIAA1429 (Abcam, UK), FTO (Abcam, UK), ALKBH5 (Abcam, UK) overnight at 4 °C. The secondary antibody (Abcam, UK) was then incubated with the membranes for 2 h at room temperature. Finally, the protein bands were visualized by ECL Western Blot detection kit (GE Healthcare Bio-science, USA) and quantified by Image J software.

### M6A-RIP–RT-PCR

The m6A-RIP test was carried out by Magna MeRIP m6A Kit (Merck, Germany) according to the manufacturer’s instructions. Total RNA was extracted and lysed in high salt lysis buffer. The RNA fragment was incubated with Magnetic beads bound by anti-M6A antibody. M6A salt was washed, eluted with RNA, purified with an RNA purification kit (Qiangen, USA) for qRT-PCR detection. The relative fold enrichment was calculated using 2^−ΔΔCt^ method.

### Cell counting kit-8 (CCK-8) assay

The RVECs cells were harvested and seeded into the 96-well plates at the density of 2 × 10^3^ per well. The high-glucose (30 mM) were then incubated with the cells for 0 h, 24 h, respectively. The CCK-8 kit was employed to measure cell proliferation according to the manufacturer’s protocol. Briefly, 10 µl of CCK-8 solution was added into each well for 4 h. After that, the plates were gently mixed and the microplate reader (Molecular Devices, USA) was used to measure the optical density (OD) values at the absorbance of 450 nm. The OD values were used to reflect the proliferation abilities of RVECs cells.

### Transwell experiment

Invasive potential of cells was determined by the Transwell assay. In brief, cells were detached, centrifuged, and resuspended in serum-free medium (1 × 10^5^ cells/mL). The apical chambers precoated with Matrigel (Corning, NY, USA) were loaded with 2 × 10^4^ cells, while the basolateral cells were added with 10% FBS-DMEM. The chambers were taken out after a 24-h incubation at 37 ℃, and the number of non-invaded cells was wiped away by cotton swabs, while the cells invaded to the basolateral chambers were stained with 0.1% crystal violet and observed under the microscope with 5 random fields included. Cell migration was determined in a similar manner without pre-coating Matrigel on the apical chambers.

### Wound healing assay

Wound healing assay was used to detect the migration of RVECs. As previously reported, the RVECs were seeded in 24-well plate, and the cell confluency reached over 90%, the scratch was made by a pipette tip. Then the cells were cultured for 24 h, and the scratches were observed with a microscope (Leica, Germany) to evaluate the cell migration ability.

### Tube formation assay

To investigate the ability of cell angiogenesis, tube formation assay was performed in vitro. Growth Factor Reduced Matrigel matrix was evenly dispersed in 24-well plates and incubated at 37 °C for 1 h. The cells were seeded on the matrix surface at a density of 1 × 10^5^ cells/well and cultured in an incubator for 24 h. Finally, the capillary-like structures were observed under Nikon inverted microscope and its branches were analyzed by image J software.

### Fluorescence in situ hybridization (FISH)

RNA FISH assay was detected using fluorescent In Situ Hybridization kit (RiboBio, China) following the manufacturer’s protocol. In brief, HRVECs were fixed in 4% polyformaldehyde for 15 min and rinsed twice with PBS. Subsequently, the penetration treatment was performed with 0.5% Triton-100. Then cells were hybridized overnight at 37 °C in darkness and humidity. The next day, the cells were washed with sodium citrate buffer and incubated in the blocking solution for 1 h at room temperature. The HRVECs were incubated with HRP-conjugated anti-biotin antibody at 4 °C overnight. Finally, images were observed and taken using a confocal microscope (Leica, Germany).

### Statistical analysis

All Statistical analysis was performed on SPSS 26.0 (NY, USA). All data were obtained from at least three independent experiments and presented as mean ± SD. Data were analyzed by Student’s *t*-test (two groups) and one-way or two-way analysis of variance (ANOVA) followed by Tukey’s multiple comparison test (more than two groups). *p* < 0.05 was considered statistically significant.

## Results

### High glucose induced YTHDC1 expression in RVECs

**Fig. 1:**
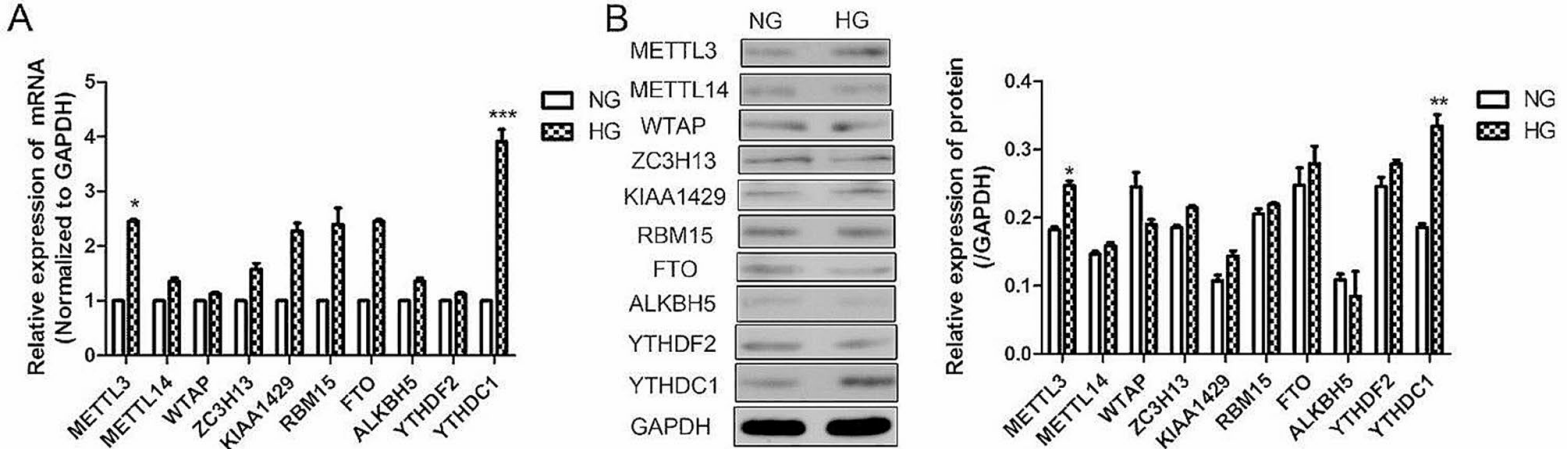
High glucose induced YTHDC1 expression in RVECs. **(A)** RT-qPCR analysis was used to detect the mRNA expression in RVECs. **(B)** western blotting assay was applied to detect the protein expressions in RVECs. The data were presented as mean ± SD, *n* = 3. NG, normal glucose; HG, high glucose. *P < 0.05, **P < 0.01, ***P < 0.001, vs. NG group

We proposed to explore the changes of m6A methylation related genes in high glucose induced RVECs. As shown in Fig. 1A, RT-*q*PCR results showed the mRNA expressions of *Ythdc1* and *Mettl3* were significantly increased in high glucose-induced RVECs compared with control group (NG). Consistent with PCR results, western blot results (Fig. 1B) that the protein expression levels of METTL3 and YTHDC1 were significantly upregulated. We hypothesized that YTHDC1 may be involved in the progression of high glucose induced diabetic retinopathy. Therefore, in our subsequent studies, we focused on the regulatory role of YTHDC1 in HG-induced RVECs.

### Silencing of YTHDC1 inhibited the proliferation, migration, invasion and tube formation ability of RVECs

**Fig. 2:**
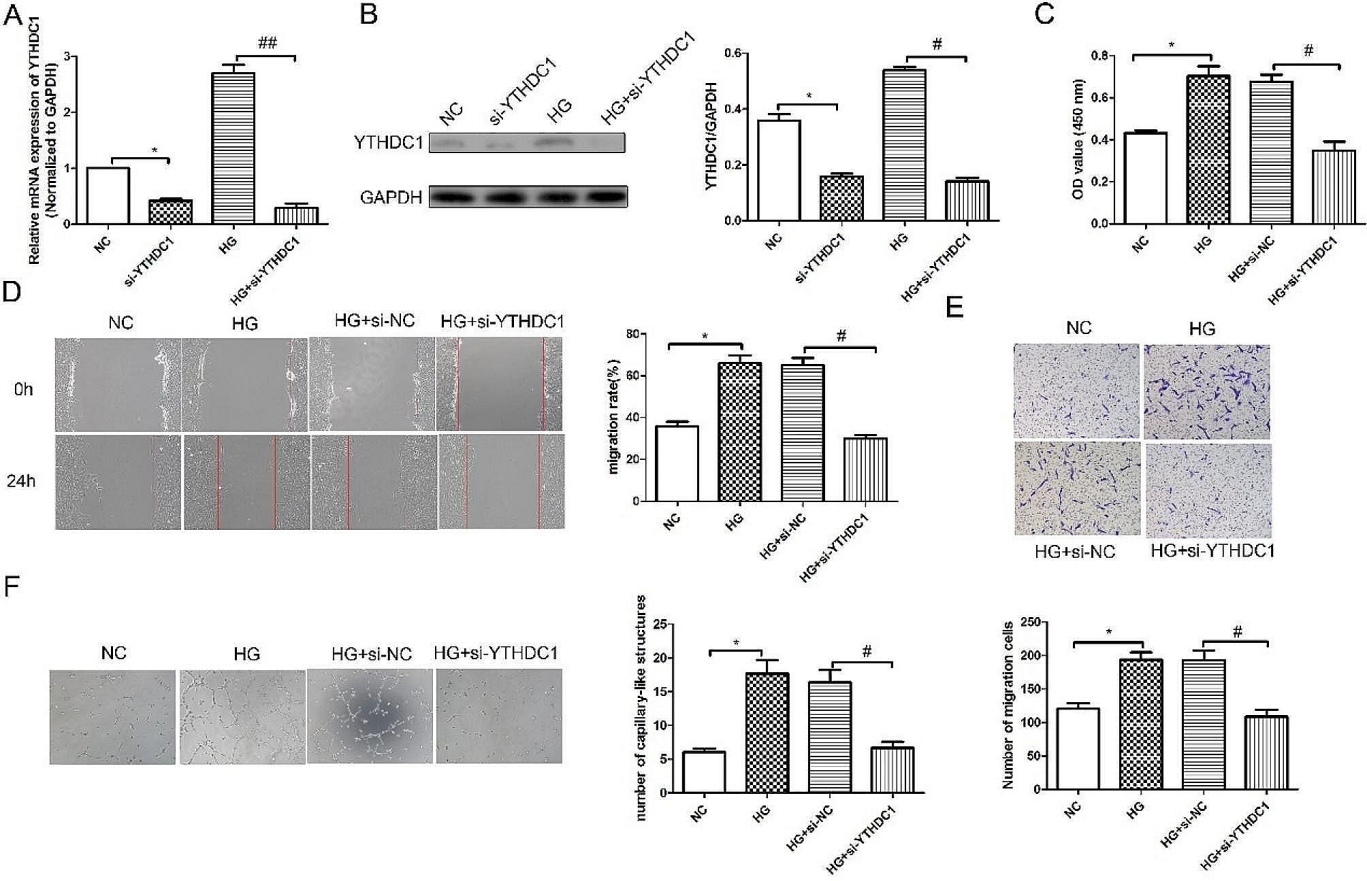
Silencing of YTHDC1 inhibited the proliferation, migration, invasion and tube formation ability of RVECs. RT-qPCR analysis **(A)** and western blot **(B)** were used to detect the mRNA and protein expression of YTHDC1 in different groups. **(C)** CCK-8 kit was used to detect the cell viability. **(D)** Wound healing assay was applied to detect the migration in different groups. **(E)** The invasion in different group was assessed using transwell experiment. **(F)** Tube formation experiment was used to detect the angiogenic ability. Data were expressed as mean ± SD, *n* = 3. ^*^P < 0.05, vs. NC group, ^#^*P* < 0.05, ^##^*P* < 0.01, vs. HG + si-NC group

To explore the biological role of YTHDC1, siRNA negative control (si-NC) or YTHDC1 siRNA were transfected into RVECs. First, RT-qPCR analysis (Fig. 2A) and western blot results (Fig. 2B) confirmed the success of transfection. Besides, CCK-8 kit results (Fig. 2C) showed that compared with NC group, the cell viability in HG group was increased significantly. While the cell viability in HG + si-YTHDC1 group was reduced obviously compared with HG + si-NC group. Wound healing assay (Fig. 2D) revealed that the migration in HG group was increased significantly compared with NC group. Compared with HG + si-NC group, the migration in HG + si-YTHDC1 group was reduced remarkedly. Transwell experiment results (Fig. 2E) showed that the invasion in HG + si-YTHDC1 group was reduced significantly compared with HG + si-NC group. Tube formation experiments results (Fig. 2F) showed that compared with HG + si-NC group, the angiogenic capacity in HG + si-YTHDC1 group was reduced significantly.

### Overexpression of YTHDC1 promoted the proliferation, migration, invasion and tube formation ability of RVECs

**Fig. 3:**
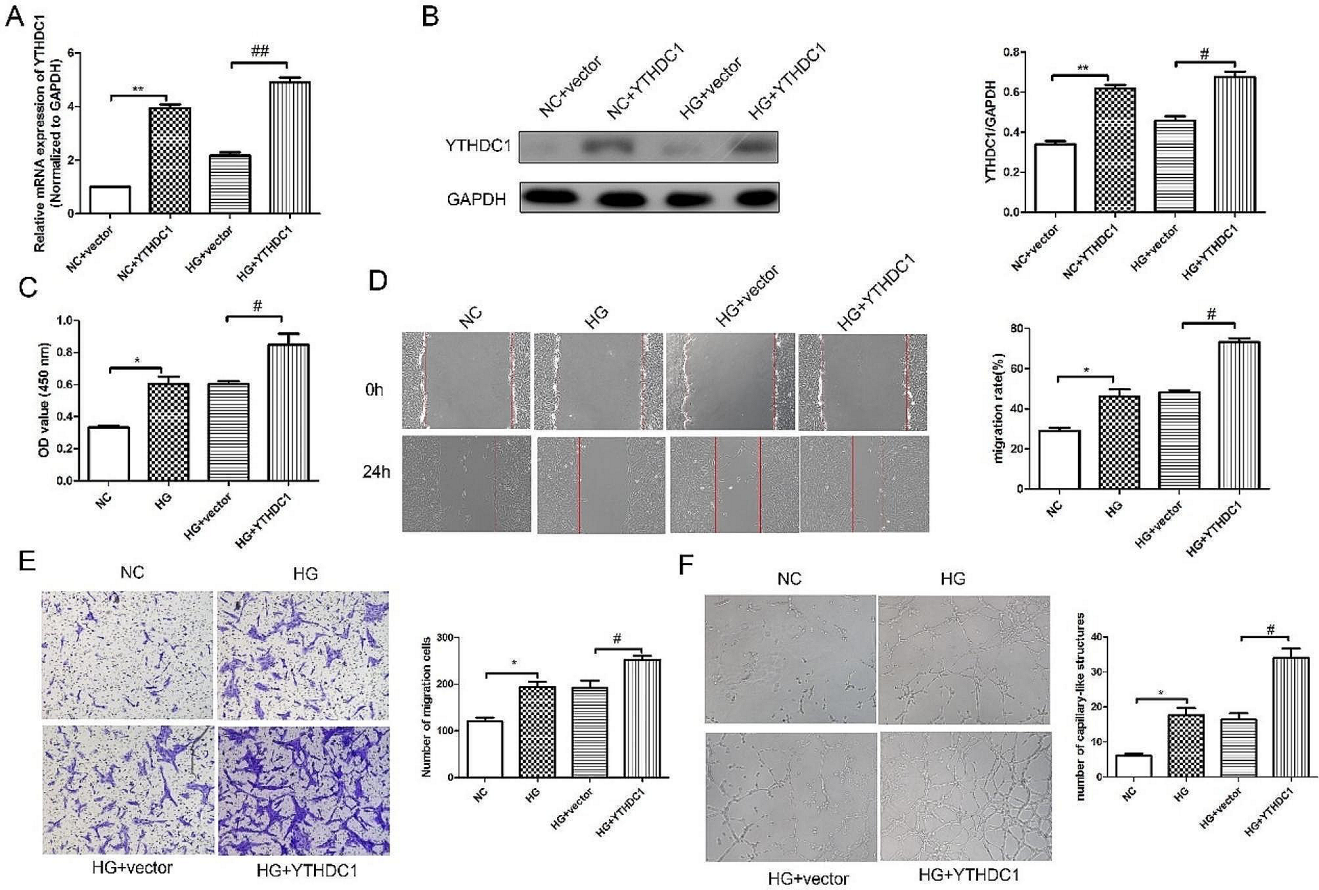
Overexpression of YTHDC1 promoted the proliferation, migration, invasion and tube formation ability of RVECs RT-qPCR analysis **(A)** and western blot **(B)** were used to detect the mRNA and protein expression of YTHDC1 in different groups. **(C)** CCK-8 kit was used to detect the cell viability. **(D)** Wound healing assay was applied to detect the migration in different groups. **(E)** The invasion in different group was assessed using transwell experiment. **(F)** Tube formation experiment was used to detect the angiogenic ability. Data were expressed as mean ± SD, *n* = 3. ^*^P < 0.05, ^**^P < 0.01, vs. NC group, ^#^*P* < 0.05, ^##^*P* < 0.01, vs. HG + vector group

In addition, we investigated the effect of overexpression of YTHDC1 on the function of RVECs cells induced by HG by transfection with overexpression plasmid. RT-qPCR analysis (Fig. 3A) and western blot results (Fig. 3B) confirmed the success of transfection. Besides, CCK-8 kit results (Fig. 3C) showed that compared with NC group, the cell viability in HG group was increased significantly. While the cell viability in HG + YTHDC1 group was increased obviously compared with HG + vector group. Wound healing results (Fig. 3D) revealed that the migration in HG group was increased significantly compared with NC group. Compared with HG + vector group, the migration in HG + YTHDC1 group was increased remarkedly. Transwell experiment results (Fig. 3E) showed that the invasion in HG + YTHDC1 group was increased significantly compared with HG + vector group. Tube formation experiment results (Fig. 3F) showed that compared with HG + vector group, the angiogenic capacity in HG + YTHDC1 group was increased significantly. The above results indicated that YTHDC1 could significantly promote HG-induced RVECs cell migration, invasion and angiogenesis.

### Si-YTHDC1 down-regulated CDK6 expression

**Fig. 4:**
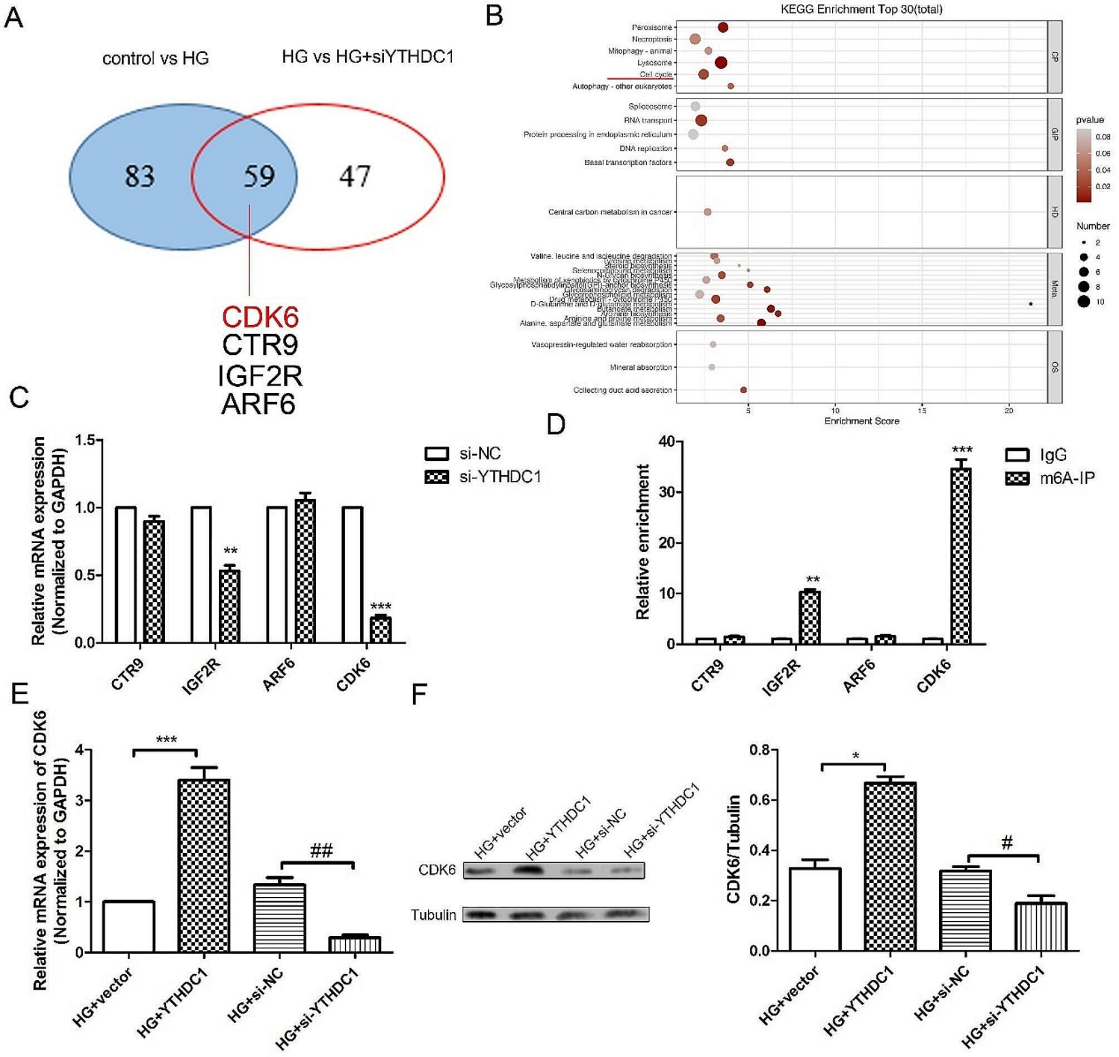
Si-YTHDC1 down-regulated CDK6 expression **(A)** m6A methylation chip was used to analyze the differential genes regulated by YTHDC1. **(B)** KEGG pathway enrichment analysis. **(C)** RT-qPCR analysis was used to detect the mRNA expressions of CTR9, IGF2R, ARF6 and CDK6. **(D)** m6A-IP-qPCR assay was used to detect the CDK6 level. RT-qPCR analysis **(E)** and western blot **(F)** were performed to detect the mRNA and protein expresions of CDK6 in different groups. The data were expressed as mean ± SD, *n* = 3. *P* < 0.05, ^**^P < 0.01, ^***^P < 0.001, vs. HG + vector group; ^#^*P* < 0.05, ^##^*P* < 0.01, vs. HG + si-NC group

Combined with the m6A methylation detection results (Fig. 4A) and data analysis, we screened CDK6, CTR9, IGF2R and ARF6 as a differential gene for study. KEGG enrichment analysis results (Fig. 4B; Table 1) showed that si-YTHDC1 mainly regulated downstream cell cycle-related signaling pathways. In the present study, RT-qPCR (Fig. 4C) confirmed that the mRNA expression levels of CDK6 and IGF2R in si-YTHDC1 group were significantly down-regulated in RVECs cells induced by high glucose compared with si-NC group. And m6A-IP-qPCR assay results (Fig. 4D) showed CDK6 and IGF2R m6A methylation was increased significantly. Given that CDK6 showed the most significant differences, we ultimately chose CDK6 as the downstream target molecule for further investigation. Besides, RT-qPCR results (Fig. 4E) showed that overexpression of YTHDC1 could up-regulate the mRNA expression of CDK6, while, silencing YTHDC1 could down-regulate the mRNA expression of CDK6 in HG-induced RVECs. Similarly, western blot results (Fig. 4F) showed the same trend.

**Table 1:** Representative differential genes in RVECs (fc < 0, down-regulated; fc > 0, up-regulated)

Gene name	Fold changes
	control vs. HG
CDK6	1.18
CBR4	0.942
SUPT16	1.09
CHORDC1	0.69
GDI1	0.712
CPD	1.16
FAM13A	1.41
ACADSB	0.77
RND3	1.22
MRP149	1.3
GM8979	-1.55
IP6K1	-0.767
SETD7	-1.75
NUP50	-1.03
NTP20	-1.64
SRCAP	-1.18
JOSD1	-2.04
MYO7B	-1.88
BHLHE40	-0.629
MRPL32	-0.746

### YTHDC1 regulated CDK6 m6A modification

**Fig. 5:**
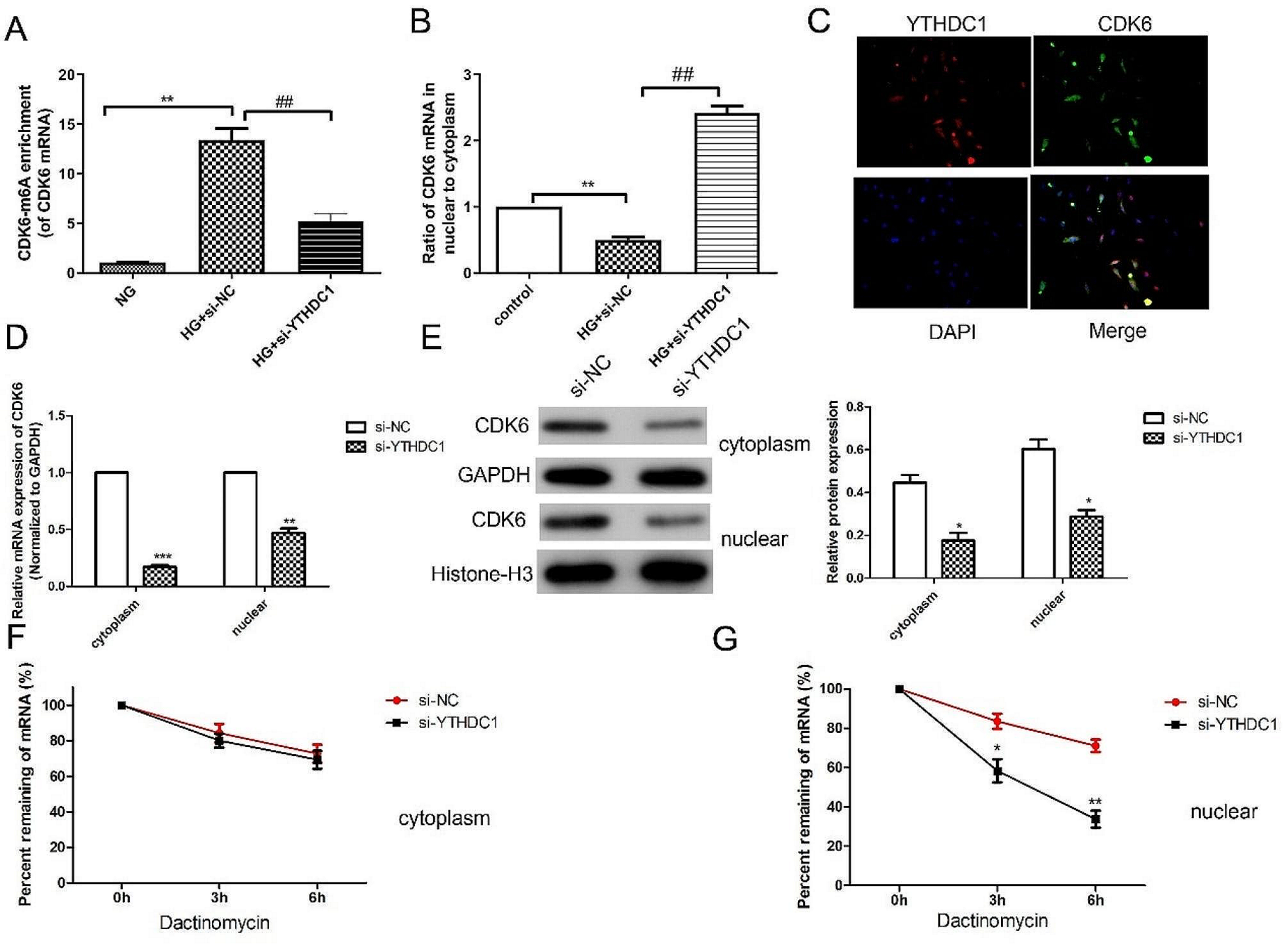
YTHDC1 regulated CDK6 m6A modification. **(A)** CDK6 m6A level in high glucose-induced RVECs was examined by RIP-qPCR **(B)** RT-qPCR analysis was used to detect the ratio of CDK6 mRNA in cytoplasm to nucleus **(C)** RNA-FISH was utilized to examine the co-localization between YTHDC1 (Cy3-labelled) and CDK6 (FITC-labelled) in the nucleus of HRVECs cells. Nuclei were stained with DAPI (blue). RT-qPCR analysis **(D)** and western blot **(E)** were used to detecet the mRNA and protein expressions of CDK6 in the cytoplasm and nuclear. The mRNA expressions of CDK6 in cytoplasm **(F)** and nuclear **(G)** were measured at different time points after actinomycin D treatment. The data were expressed as mean ± SD, *n* = 3. ^**^P < 0.01, ^***^P < 0.001, vs. NG group; ^##^*P* < 0.01, vs. si-NC group

As we known, YTHDC1 regulates gene expression through m6A modification. Here, we detected the m6A content of CDK6 by RIP-*q*PCR. As displayed in Fig. 5A, the CDK6 m6A content was increased under high glucose condition. Compared with HG + si-NC group, the CDK6 m6A methylation level in HG + si-YTHDC1 grpup decreased significantly. RT-qPCR results (Fig. 5B) showed that compared with the HG + si-NC group, the ratio of CDK6 in the nucleus to cytoplasm in HG + si-YTHDC1 group was significantly increased. FISH results (Fig. 5C) revealed that YTHDC1 and CDK6 co-locate in the nucleus. RT-qPCR results (Fig. 5D) showed that compared with the si-NC group, the mRNA expression of CDK6 was significantly down-regulated in the si-YTHDC1 group, especially in the cytoplasm. Similarly, western blot results (Fig. 5E) confirmed that CDK6 protein level showed the same trend. After actinomycin D treatment, mRNA expression of CDK6 were analyzed by RT-qPCR. As shown in Fig. 5FG, we found that the mRNA expression of CDK6 in the nucleus of the si-YTHDC1 group was significantly lower than that in the si-NC group, but there was no significant change in the cytoplasm. Si-YTHDC1 drives the degradation of CDK6 mRNA in the nucleus but does not affect the stability of CDK6 mRNA in the cytoplasm. In summary, it suggested that YTHDC1 up-regulated CDK6 expression and plays a role in the nucleus.

### Silencing CDK6 inhibited the proliferation, migration, invasion and angiogenic capacity of RVECs induced by HG

**Fig. 6:**
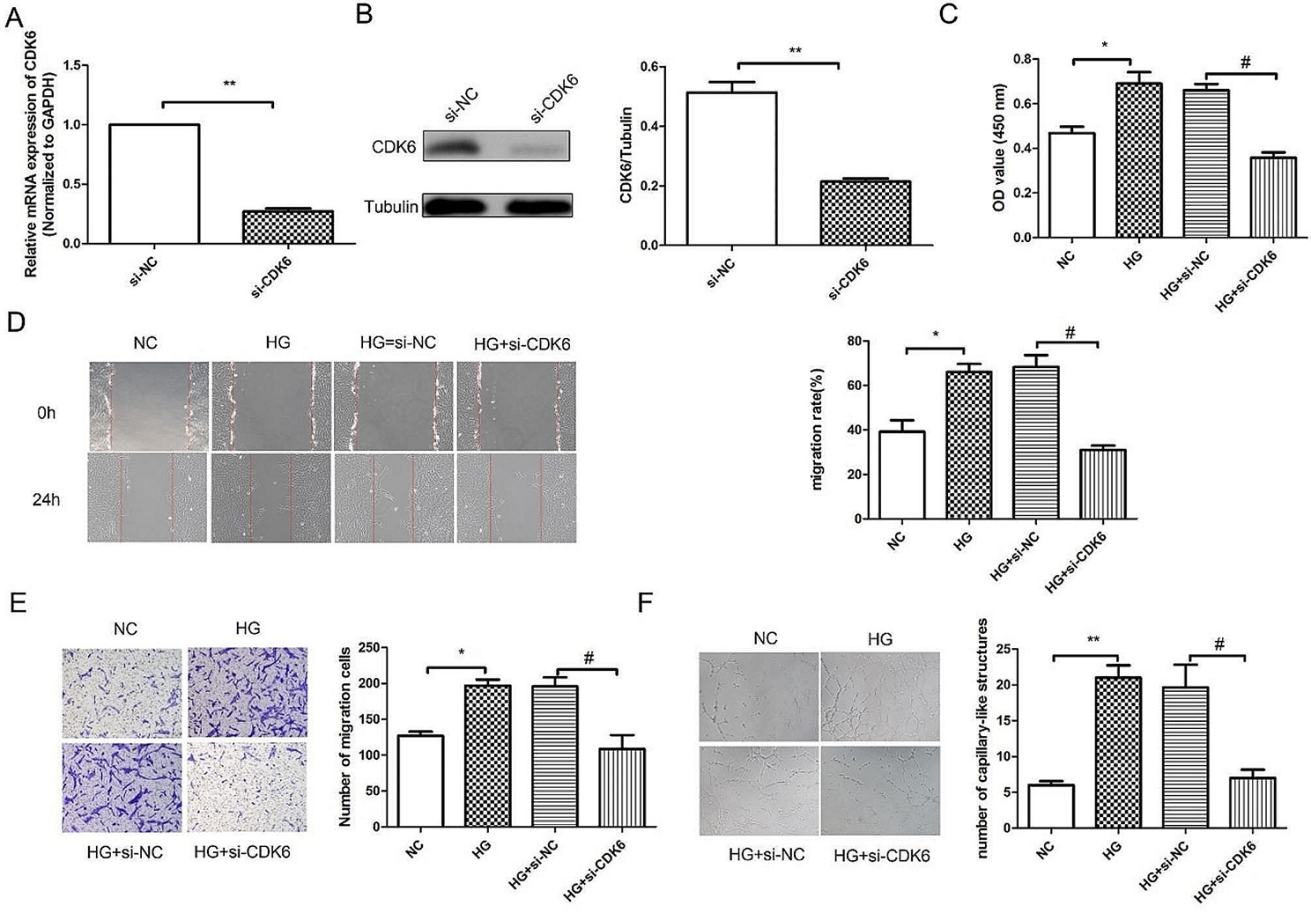
Silencing CDK6 inhibited the proliferation, migration, invasion and tube formation ability of RVECs induced by HG. RT-qPCR **(A)** analysis and western blot assay **(B)** were used to detect the mRNA and protein expression of CDK6 in RVECs. (C) CCK-8 kit was used to detect the cell viability. **(D)** Wound healing assay was used to detect the migration. **(E)** The invasion of RVECs was detected using transwell experiment. **(F)** Tube formation experiment was used to detect the angiogenic capacity in RVECs. The data were expressed as mean ± SD, *n* = 3. ^**^P < 0.01, ^***^P < 0.001, vs. NG group; ^##^*P* < 0.01, vs. si-NC group

Subsequently, the effect of CDK6 silencing on RVECs cell function was detected by wound healing assay and transwell experiment. RT-qPCR results (Fig. 6A) showed that compared with si-NC group, the mRNA expression of CDK6 was reduced significantly. Western blot results (Fig. 6B) showed the same trend as PCR results. RT-qPCR and western blot confirmed the successful transfection of si-CDK6. CCK-8 kit results (Fig. 6C) showed that compared with NC group, the cell viability in HG group was increased significantly. While the cell viability in HG + si-CDK6 group was reduced obviously compared with HG + si-NC group. Wound healing results (Fig. 6D) showed that compared with NC group, the migration in HG group was increased significantly. Compared with HG + si-NC group, the migration in HG + si-CDK6 group was reduced. In addition, transwell experiment results (Fig. 6E) showed that compared with HG + si-NC group, the invasion in HG + si-CDK6 was reduced significantly. Tube formation experiment results (Fig. 6F) showed that compared with NC group, the angiogenic capacity in HG group was increased significantly. While, compared with HG + si-NC group, the angiogenic capacity in HG + si-CDK6 group was reduced. Taken together, we suggested that silencing CDK6 inhibited the proliferation, migration, invasion and angiogenic capacity of RVECs induced by HG.

### YTHDC1 aggravated HG-induced RVECs dysfunction and rescued by CDK6

**Fig. 7:**
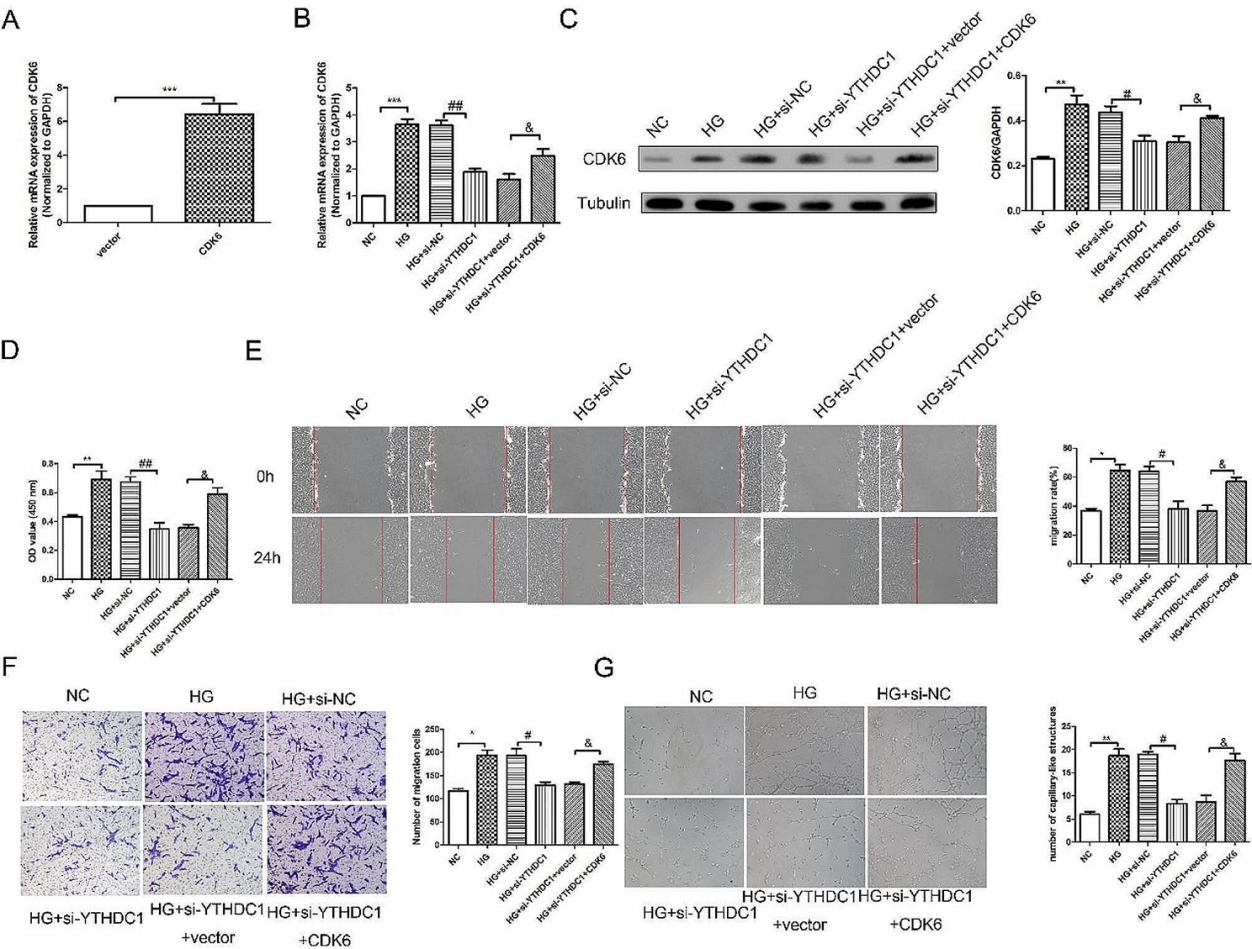
YTHDC1 aggravated HG-induced RVECs dysfunction and rescued by CDK6. **(A)** RT-qPCR analysis was used to detect the mRNA expression of CDK6. The mRNA expression **(B)** and protein expression **(C)** of CDK6 in different groups were determined using RT-qPCR analysis and western blot. **(D)** CCK-8 kit was used to detect the cell viability. **(E)** Wound healing assay was performed to detect the migration in different groups. The invasion in different groups was detected using Transwell experiment(F). **(G)** Tube formation experiment was used to detect the angiogenesis ability. The data were expressed as mean ± SD, *n* = 3,^*^P < 0.05, ^**^P < 0.01, ^***^P < 0.001, vs. NC group; ^#^*P* < 0.05, ^##^*P* < 0.01, vs. HG + NC group; ^&^*P* < 0.05, vs. HG + si-YTHDC1 + vector group

We further studied the mechanism of YTHDC1 regulating RVECs cell function. RT-qPCR results (Fig. 7A) confirmed CDK6 overexpression plasmid transfection success. RT-qPCR results (Fig. 7B) showed that compared with HG + si-YTHDC1 + vector group, the mRNA expression of CDK6 was increased in HG + si-YTHDC1 + CDK6 group. Western blot results (Fig. 7C) also showed the same trend. CCK-8 kit results (Fig. 7D) showed that compared with HG + si-YTHDC1 + vector group, the cell viability in HG + si-YTHDC1 + CDK6 group was increased. Wound healing results (Fig. 7E) showed that compared with HG + si-YTHDC1 + vector group, the migration in HG + si-YTHDC1 + CDK6 group was increased significantly. Transwell experiment results (Fig. 7F) showed that the invasion in HG + si-YTHDC1 + CDK6 group was increased significantly compared with HG + si-YTHDC1 + vector group. Tube formation experiment results (Fig. 7G) showed that the angiogenic capacity in HG + si-YTHDC1 + CDK6 group was increased significantly compared with HG + si-YTHDC1 + vector group. We suggested that YTHDC1 aggravated HG-induced RVECs dysfunction and rescued by CDK6.

### Overexpression of CDK6 reversed the protective effect of sh-YTHDC1 on diabetic retinopathy in rats

**Fig. 8:**
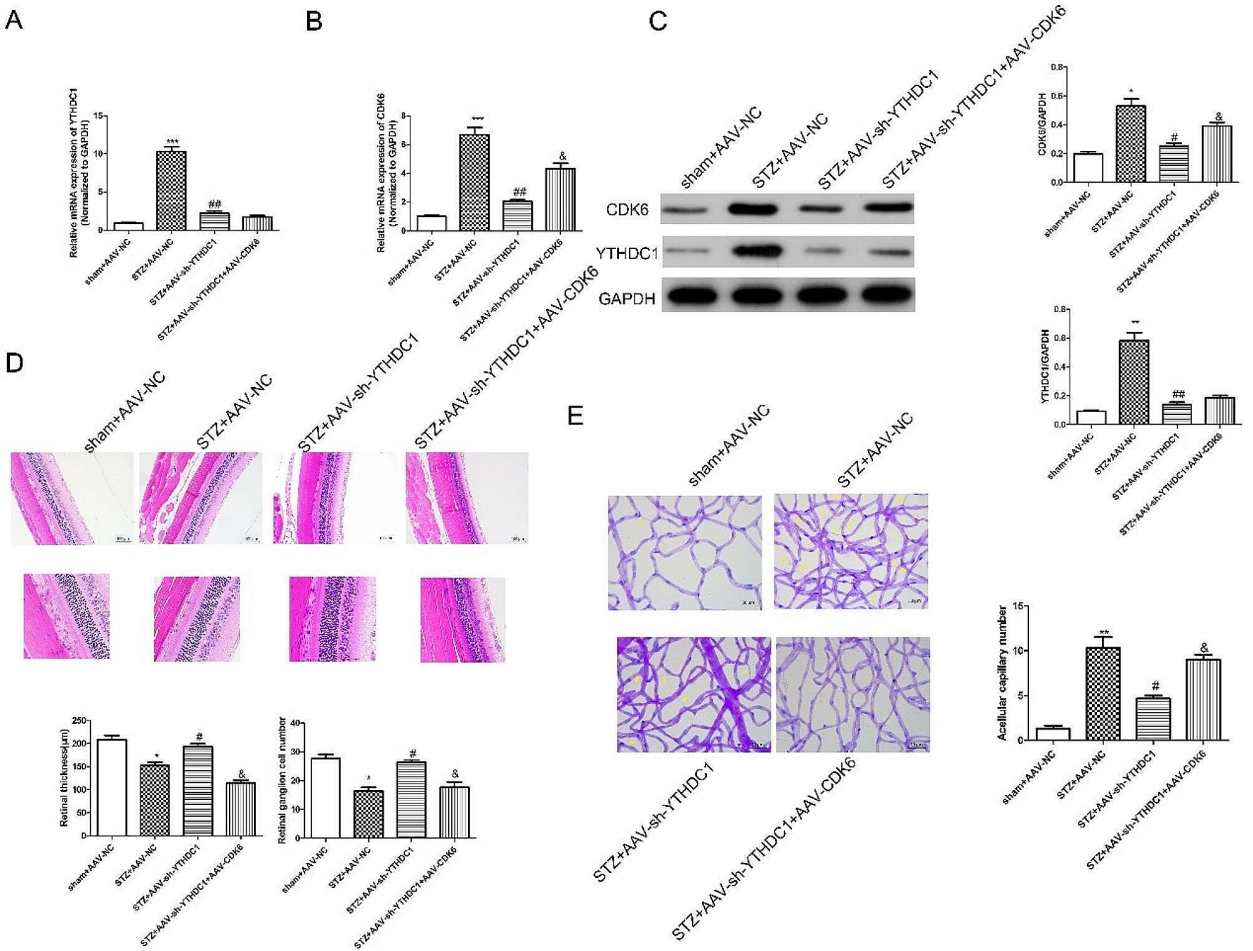
Overexpression of CDK6 reversed the protective effect of sh-YTHDC1 on diabetic retinopathy in rats. RT-qPCR analysis was used to detect the mRNA expressions of *Ythdc1***(A)** and *Cdk6***(B)** in retinal tissues. **(C)** western blot was performed to detect the protein expressions of YTHDC1 and CDK6 in retinal tissues. **(D)** The representative H&E stained images and quantitative analysis plots. **(E)** Periodic Acid-Schiff staining was used to detect the morphological changes of retinal capillaries. *P < 0.05, **P < 0.01, ***P < 0.001, vs. sham + AAV-NC group; ^#^*P* < 0.05, ^##^*P* < 0.01, vs. STZ + AAV-NC group; ^&^*P* < 0.05, vs. STZ + AAV-sh-YTHDC1 group, *n* = 6

Finally, we investigated whether overexpression of CDK6 affected the protective effect of sh-YTHDC1 in modulating diabetic retinal vascular dysfunction. RT-qPCR results (Fig. 8A) showed that compared with STZ + AAV-NC group (STZ + AAV-empty vector), the expressions of YTHDC1 was reduced significantly in STZ + AAV-sh-YTHDC1 group. As shown in Fig. 8B, compared with STZ + AAV-NC group, the expressions of CDK6 was reduced significantly in STZ + AAV-sh-YTHDC1 group. While the expression of CDK6 was higher obviously in STZ + AAV-sh-YTHDC1 + AAV-CDK6 group than that in STZ + AAV-sh-YTHDC1 group. Consistent with the RT-pPCR results, western blot results (Fig. 8C) showed the same protein trend. H&E staining was used to evaluate the pathological changes of retinopathy. It (Fig. 8D) revealed that compared with sham group, retinal thickness was significantly thinner in diabetic rats. Compared with STZ + AAV-NC group, the retinal thickness was much thicker in STZ + AAV-sh-YTHDC1 group. Comapared with STZ + AAV-sh-YTHDC1 group, the the retinal thickness was much thinner in STZ + AAV-sh-YTHDC1 + CDK6 group. Retinal trypsin digestion experiment results (Fig. 8E) showed that high glucose led to retinal capillary formation, and sh-YTHDC1 significantly improved vascular dysfunction. While, the capillary formation increased significantly in STZ + AAV-sh-YTHDC1 + CDK6 group. The results further confirmed that overexpression of CDK6 reversed the protective effect of sh-YTHDC1 on diabetic retinopathy in vivo.

## Discussion

The regulation of transcription after *N*6-methyladenosine (m6A) modification has been widely reported [18], and the study also found that methylation modification related genes in HG-induced RVECs cells were significantly up-regulated. We hypothesized that m6A methylation may be involved in key processes in the progression of retinopathy.

In this study, we found that YTHDC1 was significantly upregulated in RVECs induced by HG. Overexpression YTHDC1 can significantly promote the proliferation, invasion, migration and tubulogenesis of RVECs induced by HG, and aggravate the damage of RVECs cells. The regulatory role of YTHDC1 in many diseases [19, 20] has been widely confirmed. YTHDC1 disrupts the stability of PTEN mRNA and activates Akt phosphorylation to alleviate ischemic stroke induced neurological injury [21]. Zhu el al [22] reported YTHDC1-mediated VPS25 regulated the cell cycle by activating the JAK-STAT signaling pathway in human glioma cells. YTHDC1 promoted the cytoplasmic output of methylated circNSUN2, regulates the stability of HMGA2, and promotes liver metastasis of colorectal cancer [23]. Whereas, the molecular mechanism of YTHDC1 in diabetic retinopathy is still unclear. Hence, a large number of basic studies need to focus on the regulatory role of YTHDC1 in diabetic retinopathy.

Combined with previous report, we have confirmed that CDK6 is the target molecule of YTHDC1 downstream regulation. Our study found that overexpression of YTHDC1 significantly upregulated CDK6 expression, while silencing of YTHDC1 significantly inhibited CDK6 expression. The m6A level of CDK6 was significantly up-regulated in RVECs cells induced by high glucose, while silencing YTHDC1 significantly inhibited the CDK6 methylation. It suggested that the regulation of YTHDC1 in diabetic retinopathy may be related to the CDK6 methylation. CDK6 methylation could enhance its stability in HG-induced RVECs and promote the regulation of cell cycle and proliferation. CDK6 is a kinase catalytic subunit of a protein kinase complex that is involved in G1 process and G1/S transition [24]. Li et al [25] reported that P27 inhibited CDK6 expression, leading to cell cycle arrest and inhibiting cell proliferation. MiR-298 could target the expression of CDK6 to inhibit the proliferation of thyroid cells and promote the apoptosis of thyroid cancer cells [26]. Chen et al [27] found that miRNA-524-5p could negatively regulate CDK6 expression, inhibit osteosarcoma cell proliferation and induce cell cycle arrest.

With the development of m6A methylation technology and the further study of its role in diseases, a variety of regulatory enzymes related to m6A modification have been discovered and their regulatory mechanisms have been confirmed. As a reader protein, YTHDC1 has been shown to significantly promote CDK6 methylation in HG-induced RVECs. YTHDC1 regulated the expression of CDK6 and contributes to its proliferation, migration and tubulogenesis in RVECs cells induced by HG. We confirmed that YTHDC1 plays a key role in HG induced RVECs cells, suggesting that YTHDC1 may serve as a new drug therapeutic target for diabetic retinopathy. However, the limitation of the study was that it only confirmed that YTHDC1 regulates CDK6 methylation modification in HG-induced RVECs at the phenotypic level, without in-depth exploration CDK6 methylation sites. We will further explore the binding site of YTHDC1 methylation modification, explore other possible regulatory factors, and further understand the role of YTHDC1 in m6A methylation in retinopathy induced by HG.

## Data Availability

The datasets during the study are available from the corresponding author on reasonable request.
